# Chronic alcohol abuse affects the clinical course and outcome of community-acquired bacterial meningitis

**DOI:** 10.1007/s10096-019-03661-5

**Published:** 2019-08-07

**Authors:** Marcin Paciorek, Agnieszka Bednarska, Dominika Krogulec, Michał Makowiecki, Justyna D Kowalska, Dominik Bursa, Anna Świderska, Joanna Puła, Joanna Raczyńska, Agata Skrzat-Klapaczyńska, Magdalena Zielenkiewicz, Marek Radkowski, Tomasz Laskus, Andrzej Horban

**Affiliations:** 1 Department of Adult Infectious Diseases, Medical University of Warsaw, Warsaw, Poland; 2 Medical University of Warsaw, Warsaw, Poland; 3 Institute of Mathematics, University of Warsaw, Warsaw, Poland; 4 Department of Immunopathology of Infectious and Parasitic Diseases, Medical University of Warsaw, Warsaw, Poland

**Keywords:** Bacterial meningitis, Alcoholism, Outcome, SOFA

## Abstract

The aim of the study was to determine the effect of chronic alcohol abuse on the course and outcome of bacterial meningitis (BM). We analyzed records of patients with BM who were hospitalized between January 2010 and December 2017 in the largest neuroinfection center in Poland. Out of 340 analyzed patients, 45 (13.2%) were alcoholics. Compared with non-alcoholics, alcoholics were more likely to present with seizures (*p* < 0.001), scored higher on the Sequential Organ Failure Assessment (SOFA) (*p* = 0.002) and lower on the Glasgow Coma Scale (GCS) (*p* < 0.001), and had worse outcome as measured by the Glasgow Outcome Score (GOS) (*p* < 0.001). Furthermore, alcoholics were less likely to complain of headache (*p* < 0.001) and nausea/vomiting (*p* = 0.005) and had lower concentration of glucose in cerebrospinal fluid (CSF) (*p* = 0.025). In the multiple logistic regression analysis, alcoholism was associated with lower GCS (*p* = 0.036), presence of seizures (*p* = 0.041), male gender (*p* = 0.042), and absence of nausea/vomiting (*p* = 0.040). Furthermore, alcoholism (*p* = 0.031), lower GCS score (*p* = 0.001), and higher blood urea concentration (*p* = 0.018) were independently associated with worse outcome measured by GOS. Compared with non-alcoholics, chronic alcohol abusers are more likely to present with seizures, altered mental status, and higher SOFA score and have an increased risk of unfavorable outcome. In multivariate analysis, seizures and low GCS were independently associated with alcoholism, while alcoholism was independently associated with worse outcome.

**Electronic supplementary material:**

The online version of this article (10.1007/s10096-019-03661-5) contains supplementary material, which is available to authorized users.

## Introduction

In Poland, where the registration of bacterial meningitis (BM) cases is mandatory, the annual incidence of BM in the years 2010–2017 ranged between 1.97/100,000 and 2.5/100,000 [1–3] which is higher than is some other European countries such as Finland (0.7/100,000), Netherlands (0.94/100,000), or England and Wales (1.44/100,000) [4–6]. Alcohol per capita consumption (in liters of pure alcohol) for population ≥ 15 years old was 11.4 in 2010 and 11.6 in 2016 which is similar to many other European countries [7]. Alcoholics are more susceptible to bacterial infections and these infections carry a worse prognosis. There is evidence that the relative risk of bacterial pneumonia correlates with the level of alcohol intake [8]. While this may be in part a consequence of lifestyle and malnutrition, there is evidence that chronic alcohol abuse itself may impair various host immune responses [9, 10]. The aim of our study was to determine whether chronic alcohol abuse has any impact on clinical manifestations, etiologic factors, and outcome in BM. Such an analysis is rare in the literature and was confined so far to two studies from Netherlands [11, 12].

## Materials and methods

We evaluated records of adult (≥ 18 years old) patients with community-acquired BM who were admitted to the Hospital for Infectious Diseases in Warsaw from January 1, 2010, to December 31, 2017. The diagnosis of BM was based on fulfilling at least one of the following criteria: positive cerebrospinal fluid (CSF) culture, positive CSF Gram staining, and typical CSF findings (pleocytosis ≥ 100 cells/μL with ≥ 90% neutrophils and decrease of CSF glucose level < 2.2 mmol/L). Patients with CSF findings typical for BM but negative blood and CSF culture and negative microscopic CSF examination were considered to have BM of unknown etiology. Diagnosis of tuberculous meningitis was based on at least one of the following: positive culture, positive nucleic acid amplification, and positive Ehrlich-Ziehl-Neelsen staining of CSF. Glasgow Coma Scale (GCS) and Sequential Organ Failure Assessment (SOFA) scores were calculated at admission, while Glasgow Outcome Score (GOS) was assessed at the time of discharge from hospital.

Initial antimicrobial treatment followed current guidelines [13]. Patients with meningitis secondary to head trauma, neurosurgical procedures, and hospital-acquired infections were excluded from analysis.

Alcoholism was diagnosed according to the World Health Organization (WHO) criteria [14]. Questions about potential alcohol abuse were a standard part of medical interview, and information was obtained from patients and/or their relatives.

The Mann-Whitney *U* test was used to compare continuous variables, and the chi-square test was used to evaluate nominal variables. Logistic regression was used to calculate adjusted odds ratios and to determine variables independently associated with alcoholism and those independently influencing outcome reflected by GOS. Since GOS is not a nominal but continuous variable, we used the general linear model to calculate the coefficients for this analysis. Statistical analyses were performed using program R version 3.5.2 [15].

## Results

The final analysis included 340 patients with bacterial meningitis (211 men and 129 women, median age 57, interquartile range [IQR] 41–69). Among this group, 45 (13.2%) patients were considered alcoholics (39 men and 6 women, median age 53, IQR 44–59).

At admission, alcoholic patients were more likely to present with seizures (33.3% vs 12.6%, *p* < 0.001) (Table 1) but were less likely to complain of headache (23.3% vs 52.3%, *p* < 0.001) and nausea/vomiting (11.4% vs 33.6%, *p* = 0.005). Furthermore, they scored higher on the SOFA (median 3 [IQR 2–6] vs median 2 [IQR 1–5], *p* = 0.002) and lower on the GCS (median 10 [IQR 7–12] vs median 12 [IQR 9–14], *p* < 0.001). Alcoholic patients were also more likely to require intensive care unit (ICU) admission (48.9% vs 37.6%), and their mortality was higher (24.4% vs 15.4%), but these differences did not reach statistical significance. The clinical outcome reflected by the GOS was significantly worse among alcoholics (median 3 [IQR 1–5] vs median 5 [IQR 3–5], *p* < 0.001; Table 1).

**Table 1:** Demographic, clinical, and etiological data in alcoholic and non-alcoholic patients with bacterial meningitis

	Alcoholics *n* = 45	Non-alcoholics *n* = 295	*p* value
Patient characteristics before and on admission*			
Age (years)	53 (44–59)	58 (39–70)	0.124
Male (%)	39/45 (86.7)	172/295 (58.3)	*< 0.001*
Headache (%)	10/43 (23.3)	146/279 (52.3)	*< 0.001*
Fever ≥ 37.8 °C (%)	31/45 (68.9)	235/285 (82.4)	0.052
Glasgow Coma Scale score	10 (7–12)	12 (9–14)	*< 0.001*
Neck stiffness (%)	38/45 (84.4)	221/283 (78.1)	0.597
Nausea/vomiting (%)	5/44 (11.4)	97/289 (33.6)	*0.005*
Seizures (%)	15/45 (33.3)	37/294 (12.6)	*< 0.001*
Ataxia (%)	2/43 (4.4)	13/289 (4.5)	0.987
Aphasia (%)	4/41 (8.9)	25/286 (8.7)	0.974
Cranial nerve paralysis (%)	2/45 (4.4)	20/293 (6.8)	0.547
Hemiparesis (%)	9/45 (20)	31/293 (10.6)	0.069
Vertebral pain/back pain (%)	5/44 (11.4)	29/281 (10.3)	0.834
Skin rash (%)	2/45 (4.4)	21/285 (7.4)	0.474
Disease severity/outcome			
SOFA score on admission	3 (2–6)	2 (1–5)	*0.002*
Requiring ICU admission (%)	22/45 (48.9)	111/295 (37.6)	0.154
Glasgow Outcome Score	3 (1–5)	5 (3–5)	*< 0.001*
Mortality (%)	11/45 (24.4)	45/292 (15.4)	0.130
Identified pathogen (%)			
*Streptococcus pneumoniae*	8/45 (17.8)	58/295 (19.7)	0.766
*Staphylococcus*	4/45 (8.9)	37/295 (12.5)	0.483
*Neisseria meningitidis*	5/45 (11.1)	25/295 (8.5)	0.561
*Listeria monocytogenes*	0/45 (0)	24/295 (8.14)	0.094
*Mycobacterium tuberculosis*	3/45 (6.7)	17/295 (5.8)	0.810
Other Gram-positive	6/45 (13.3)	17/295 (5.8)	0.060
Other Gram-negative	1/45 (2.2)	11/295 (3.7)	0.610
*Haemophilus influenzae*	0/45 (0)	4/295 (1.4)	0.432
Unknown	18/45 (40)	102/295 (34.6)	0.478
Diagnostic LP after antibiotic treatment initiation	26/39 (66.7)	134/245 (54.7)	0.161

*Data are presented as median (interquartile range) or *n/N* (%), *p* values < 0.05 are italicized

*LP* lumbar puncture, *SOFA* Sequential Organ Failure Assessment score, *ICU* intensive care unit

Analysis of laboratory parameters (Table 2) revealed that alcoholic patients had significantly higher serum concentration of d-dimers (median 3273 μg/L [IQR 1751–5817] vs median 2241 μg/L [IQR 1159–4262], *p* = 0.013) and lower concentration of glucose in CSF (median 0.58 mmol/L [IQR 0–2.3] vs median 1.97 [IQR 0.11–3.40], *p* = 0.025; Table 2).

**Table 2:** Laboratory blood and cerebrospinal fluid (CSF) results in alcoholic and non-alcoholic patients with bacterial meningitis

	Alcoholics	Non-alcoholics	*p* value
Blood test results*			
CRP (mg/L)	208.5 (76.5–327)	218.5 (68–328)	0.750
Lactic acid (mmol/L)	1.91 (1.45–2.86)	2.00 (1.58–2.93)	0.822
WBC (1000 cells/μL)	14.5 (10.1–19.1)	14.3 (10.1–20.0)	0.827
PLT (1000 cells/μL)	188 (91–287)	189 (138–247)	0.986
PCT (ng/mL)	3.20 (0.46–8.88)	3.33 (0.39–13.2)	0.958
d-dimers (μg/L)	3273 (1751–5817)	2241 (1159–4262)	*0.013*
Creatinine (μmol/L)	63 (55–91)	68 (55–85)	0.533
Urea (mmol/L)	6.02 (4.47–9.72)	6.14 (4.40–10.29)	0.840
CSF test results			
Cytosis (cells/μL)	539 (259–4480)	1110 (243–3880)	0.846
Granulocytes (%)	88.5 (54–95)	87.5 (70–95)	0.838
Protein (g/L)	3.67 (1.81–7.28)	2.83 (1.33–6.36)	0.178
Glucose (mmol/L)	0.58 (0–2.3)	1.97 (0.11–3.40)	*0.025*
Lactic acid (mmol/L)	7.2 (5.6–12.2)	5.5 (3.0–10.7)	0.118
Chlorides (mmol/L)	112 (109–122)	117 (113–121)	0.101

*Data are presented as median (interquartile range), *p* values < 0.05 are italicized

*CRP* C-reactive protein, *WBC* white blood cells, *PLT* platelets, *PCT* procalcitonin, *CSF* cerebrospinal fluid

In multiple logistic regression analysis (Table 3), alcoholism was independently associated with a lower GCS score (OR 0.716, 95% CI 0.523–0.980, *p* = 0.036), male gender (OR 4.617, 95% CI 1.060–20.113, *p* = 0.042), the presence of seizures (OR 4.580, 95% CI 1.065–19.706, *p* = 0.041), and the absence of nausea/vomiting (OR 0.205, 95% CI 0.045–0.930, *p* = 0.040). Furthermore, alcoholism, lower GCS score, and higher urea blood concentration were independently associated with worse prognosis as assessed by GOS (Table 4).

**Table 3:** Multiple logistic regression analysis of factors independently associated with alcoholism in patients with bacterial meningitis

Variable*	*p* value	OR	95% CI
GCS	*0.036*	0.716	0.523–0.980
GOS	0.780	0.933	0.575–1.515
SOFA	0.075	0.814	0.649–1.021
CSF glucose	0.335	0.850	0.611–1.183
Male gender	*0.042*	4.617	1.060–20.113
Headache	0.316	0.535	0.157–1.819
Nausea/vomiting	*0.040*	0.205	0.045–0.930
Seizures	*0.041*	4.580	1.065–19.706

*Results are presented as odds ratio (OR) and confidence interval (CI), *p* values < 0.05 are italicized

*GCS* Glasgow Coma Scale, *GOS* Glasgow Outcome Scale, *SOFA* Sepsis-related Organ Failure Assessment score, *CSF* glucose concentration of glucose in cerebrospinal fluid

**Table 4:** Multiple logistic regression analysis of factors independently associated with lower Glasgow Outcome Score (GOS) in patients with bacterial meningitis

Variable	Regression coefficient	95% CI	*p* value
Alcoholism	− 0.636	− 1.209–− 0.063	*0.031*
Seizures	0.180	− 0.037–0.728	0.521
Etiology unknown	0.100	− 0.353–0.552	0.667
*Streptococcus pneumoniae*	− 0.010	− 0.669–0.470	0.732
*Neisseria meningitidis*	0.512	− 0.244–1.267	0.186
Age	− 0.009	− 0.021–0.003	0.161
GCS	0.144	0.061–0.226	*0.001*
SOFA	− 0.068	− 0.160–0.025	0.154
CRP	0.001	− 0.001–0.002	0.841
PLT	0.001	− 0.001–0.002	0.633
PCT	− 0.001	− 0.008–0.005	0.665
Urea	− 0.052	− 0.095–− 0.009	*0.018*
CSF protein	− 0.007	− 0.040–0.025	0.656
CSF glucose	0.034	− 0.061–0.129	0.484

*CI* confidence interval, *GCS* Glasgow Coma Scale, *SOFA* Sepsis-related Organ Failure Assessment score, *PLT* platelet level, *PCT* concentration of procalcitonin in blood, *CSF protein* concentration of protein in cerebrospinal fluid, *CSF glucose* concentration of glucose in cerebrospinal fluid, *p* values < 0.05 are italicized

An etiological factor was identified only in 60% of alcoholics and 65% of non-alcoholics (Table 1). However, in 67% of alcoholics and in 55% of non-alcoholics, antibiotic treatment initiation preceded diagnostic spinal tap.

## Discussion

According to national survey data [16], alcoholics constitute approximately 2% of the Polish general adult population, but among our patients with BM, this proportion was 13% which might be a direct consequence of impaired immune response to bacterial pathogens related to chronic alcohol abuse [17–20]. In the nationwide cohort study conducted in the Netherlands, the proportion of alcoholics in BM patients was also higher than in general population [7, 11].

Compared with non-alcoholics, alcohol abusers were less likely to present with fever, headache, and nausea. The absence of such typical symptoms of BM [21] might cause diagnostic problems and in effect result in the delay of treatment initiation. Another difference in the clinical presentation was higher incidence of seizures among alcoholic patients (33% vs 13%, *p* < 0.001). While high incidence of seizures among alcohol abusers presenting with BM is a well-known phenomenon, the numbers in previous reports were lower and ranged from 13 to 18% [11, 12]. Furthermore, in our multivariate analysis, the presence of seizures was independently associated with alcoholism but not with outcome.

The presence of seizures and other signs and symptoms associated with the alcohol withdrawal syndrome or even alcohol intoxication itself could also negatively affect consciousness level: in our study, alcoholic patients scored significantly lower on the GCS scale at admission compared with non-alcoholics. These findings are compatible with previous studies of van Veen et al. [11] and Weisfelt et al. [12].

While alcohol abuse was not an independent predictor for mortality in multivariate analysis, it was associated with worse outcome as measured by the GOS. These results are similar to those reported by Wiesfelt et al. [22] who also found that alcoholism is associated with unfavorable outcome but not with higher mortality.

In addition to alcohol, low GCS score and high blood urea levels were independently associated with worse outcome (Table 4). The influence of GCS on outcome is not surprising and was previously reported by others [23, 24], while increased urea might identify a subset of patients with multiple organ failure. With the exception of blood d-dimer levels and CSF glucose levels, no differences were found in the results of routine tests between alcoholic and non-alcoholic patients. High blood d-dimer levels are common in chronic alcohol abusers and could be the effect of hemostatic system activation related to oxidative stress [25]. Interestingly, low concentration of glucose in the CSF was previously correlated with adverse clinical outcomes in patients with BM [26].

*Streptococcus pneumoniae* was the most common causative microorganism (19.4%) in our patients (Table 1), but its prevalence was not as high as in some other European studies [27–29]. Such relatively low prevalence of *Streptococcus pneumoniae* is not a local phenomenon confined to our center, as it accounted for only 22% of BM cases registered in Poland [2].

In conclusion, we found that alcoholic patients with BM, when compared with their non-alcoholic counterparts, are more likely to present with seizures and more severely altered mental status and have an increased risk for unfavorable outcome.

## Electronic supplementary material

ESM 1 (PDF 2217 kb)
